# Insulin-like growth factor-1 levels are associated with high comorbidity of metabolic disorders in obese subjects; a Japanese single-center, retrospective-study

**DOI:** 10.1038/s41598-022-23521-1

**Published:** 2022-11-22

**Authors:** Haremaru Kubo, Shojiro Sawada, Michihiro Satoh, Yoichiro Asai, Shinjiro Kodama, Toshihiro Sato, Seitaro Tomiyama, Junro Seike, Kei Takahashi, Keizo Kaneko, Junta Imai, Hideki Katagiri

**Affiliations:** 1 Department of Diabetes and Metabolism, Tohoku University Graduate School of Medicine, Tohoku University Hospital, Sendai, Miyagi Japan; 2 Division of Metabolism and Diabetes, Faculty of Medicine, Tohoku Medical and Pharmaceutical University, Sendai, Japan; 3 Division of Public Health, Hygiene and Epidemiology, Faculty of Medicine, Tohoku Medical and Pharmaceutical University, Sendai, Japan

**Keywords:** Diabetes, Metabolic syndrome, Obesity, Pre-diabetes

## Abstract

Insulin like growth factor-1 (IGF-1) plays important roles in metabolic functions, especially in adulthood. Additionally, obese subjects are reportedly predisposed to having low absolute IGF-1 levels. However, the prevalence and clinical characteristics of obese subjects with low IGF-1 levels are unknown. We examined 64 obese subjects with a body mass index (BMI) ≥ 35 kg/m^2^, with no history of endocrinological disorders, receiving inpatient care. IGF-1 levels were interpreted based on the IGF-1 standard deviation score (SDS) clinically used and standardized by age and sex (low IGF-1 group; ≤ − 2.0 SDS and standard IGF-1 group; − 2.0 < and <  + 2.0 SDS). Notably, 26.6% of the subjects had low IGF-1. Body fat mass and percentage, but not BMI, were significantly higher in the low than in the standard IGF-1 group. Furthermore, natural log-transformed high-sensitivity C-reactive protein, and the frequencies of dyslipidemia and hyperuricemia were higher in the low IGF-1 group. Moreover, among the subjects without diabetes, fasting glucose levels were significantly higher in the low IGF-1 group. Stepwise variable selection procedure revealed body fat percentage to be a parameter most strongly associated with low IGF-1. Thus, low IGF-1 levels may be an important marker of adiposity-associated metabolic disorders in obese patients.

## Introduction

Insulin-like growth factor-1 (IGF-1) is an anabolic hormone mainly secreted from the liver and its synthesis and secretion depend on growth hormone (GH) produced by the pituitary^1–3^. This relationship constitutes the GH-IGF-1 axis, which has important roles in glucose and lipid metabolism^4^, body composition^5,6^, aging^7^ and malignancy development^8^. However, since GH release is pulsatile and its blood levels fluctuate markedly, a single measurement of a random GH level is minimally useful in clinical settings. On the other hand, since levels of IGF-1, unlike those of GH, are stable throughout the day due to the relatively long half-time of IGF-1, the levels are used to evaluate GH-IGF-1 status in clinical practice^9,10^. Although absolute levels of IGF-1 were employed in most previous studies, serum IGF-1 levels are widely recognized as being influenced by age and sex^7^ even in healthy subjects. Therefore, IGF-1 levels should be adjusted for age and sex and the standard deviation score (SDS) of IGF-1 was developed to evaluate the GH-IGF-1 axis^11^. Particularly, an IGF-1 SDS below − 2.0 (≤ − 2.0 SDS) means the bottom 2.5% of the population, and is thus utilized for assessments of GH-IGF-1 axis dysfunction. However, since the SDS conversion criteria were only recently established, reports using the IGF-1 SDS have been limited.

Recently, obesity has become a major public health concern worldwide^12^. Along with the increase in obese patients, the association of IGF-1 levels with obesity has been attracting attention. Several studies have shown negative correlations between increased body mass index (BMI) and absolute IGF-1 levels^6,13,14^ and several explanations for this correlation have been considered. First, obesity-related GH resistance in the liver, such as that characteristic of fatty liver, reduces IGF-1 production, leading to decreased serum IGF-1 levels^15^. Second, decreased ghrelin secretion in obese subjects may blunt GH secretion from the pituitary, resulting in low IGF-1 levels^16^. Third, hyperinsulinemia with obesity downregulates IGF-1 binding proteins (IGF-BPs), which in turn suppresses circulating IGF-1 via negative feedback^17^. However, conflicting results, i.e. normal or elevated IGF-1 levels in obese patients, were also reported^18^. Therefore, whether IGF-1 levels are positively or negatively associated with BMI remains unclear. Moreover, the prevalence of patients with low IGF-1 levels among obese subjects, as assessed with the recently established criterion defining low IGF-1 levels, IGF-1 SDS ≤ − 2.0, remains unknown. Therefore, the characteristics of obese patients with low IGF-1 levels appear to have clinical relevance. Herein, we examined the distribution of IGF-1 SDS values in Japanese subjects with BMI ≥ 35 kg/m^2^, since this level of obesity is known to be a risk factor for a range of diseases in the Japanese population^19,20^. We also analyzed the clinical characteristics of obese subjects with low IGF-1 levels as compared to obese subjects without low IGF-1 levels.

## Results

### Distribution of the IGF-1 SDS values in obese subjects with BMI ≥ 35 kg/m^2^

**Figure 1:**
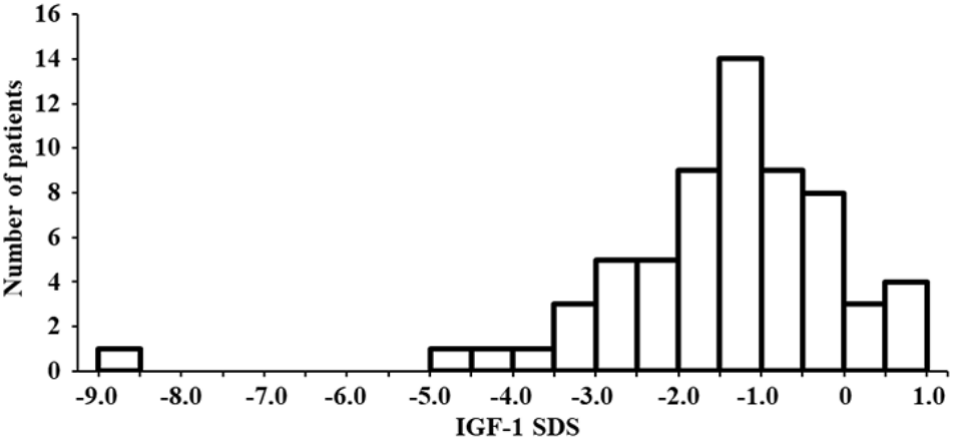
Histogram showing the number of the patients per IGF-1 SDS category. *IGF-1* insulin like growth factor-1, *SDS* standard deviation score.

A histogram illustrating the numbers of subjects with BMI ≥ 35 kg/m^2^ according to IGF-1 SDS is shown in Fig. 1. Interestingly, most (89.1%) of the subjects had IGF-1 SDS below 0, i.e. below the average of the general population matched for age and sex. The IGF-1 SDS values peaked between − 1.5 and − 1.0. In particular, no subjects had clinically elevated IGF-1 levels (≥ + 2.0 SDS). On the other hand, a considerable proportion of the subjects, 17 of 64 (26.6%), had low IGF-1 levels (i.e. IGF-1 SDS ≤ − 2.0, representing the bottom 2.5% of the general population based on the definition^11^). Taking advantage of the SDS values, we found that the subjects in this study overall showed a clear tendency for lower IGF-1 levels. In addition, patients with low IGF-1 levels were highly concentrated among those with BMI ≥ 35 kg/cm^2^.

### Clinical characteristics of obese subjects with low IGF-1 SDS

We next examined clinical characteristics of obese subjects with low IGF-1 levels. We compared metabolic and clinical parameters between subjects with and without low IGF-1 levels (IGF-1 SDS ≤ − 2.0: the low IGF-1 group). Since all subjects without low IGF-1 levels were in the − 2.0 < IGF-1 SDS < 2.0 range, we refer to this population as the standard IGF-1 group. Contrary to our expectations, there were no statistically significant differences in body weight or BMI between the two groups (Table 1). Interestingly, however, body fat mass (57.6 [50.7, 64.8] vs. 45.8 [42.2, 55,4] kg, *P* = 0.043) and body fat percentage (53.5 [50.7, 54.5] vs. 46.9 [41.9, 49.9]%, *P* = 0.0026) were significantly higher in the low IGF-1 group (Table 1) (Data hereafter shown as low IGF-1 vs. standard IGF-1 group with *P*-value). Visceral and subcutaneous adipose tissue areas evaluated by computed tomography (CT) in the low IGF-1 group tended to be higher and lower, respectively, than those in the standard IGF-1 group, although the differences were not statistically significant. Thus, despite similar BMI, the low IGF-1 group showed higher adiposity than the standard IGF group.

**Table 1:** Clinical parameters in low IGF-1 and standard IGF-1 groups.

	Low IGF-1 group (n = 17)	Standard IGF-1 group (n = 47)	*P* value	All subjects (n = 64)
Sex (male/female)	5/12	21/26	0.39	26/38
Age (years)	39.0 [30.0, 43.0]	40.0 [33.5, 55.0]	0.39	39.0 [32.8, 52.0]
IGF-1 (ng/mL)	72.0 [47.0, 91.0]	134.0 [118.0, 156.5]	< 0.001	124.0 [91.0, 143.0]
IGF-1 SDS	− 2.7 [− 3.4, − 2.4]	− 0.9 [− 1.3, − 0.3]	< 0.001	− 1.3 [− 2.0, − 0.6]
GH (ng/mL)	0.4 [0.3, 0.5]	0.5 [0.1, 0.7]	0.42	0.14 [0.08, 0.53]
Body weight on admission (kg)	106.2 [96.1, 126.0]	109.1 [97.1, 120.1]	0.82	108.8 [96.8, 121.7]
Body weight at discharge (kg)	100.6 [92.1, 115.5]	103.1 [91.7, 115.2]	0.85	101.6 [91.8, 115.3]
BMI (kg/m^2^)	41.5 [37.1, 49.7]	40.0 [37.1, 45.5]	0.27	40.2 [37.1, 46.5]
Systolic blood pressure (mmHg)	136.5 [125.5, 146.5]	133.6 [122.0, 146.0]	0.60	135.7 [124.5, 146.0]
Diastolic blood pressure (mmHg)	83.2 [74.0、97.0]	84.2 [77.0, 90.0]	0.80	83.9 [77.0, 91.5]
Body fat mass (kg)	57.6 [50.7, 64.8]	45.8 [42.2, 55.4]	0.043	50.3 [43.4, 58.35]
Body fat percentage (%)	53.5 [50.7, 54.5]	46.9 [41.9, 49.9]	0.0026	48.0 [43.4, 58.4]
Skeletal muscle mass index (kg/m^2^)	10.8 [10.5, 13.1]	11.6 [10.7, 12.2]	0.78	11.6 [10.7, 12.4]
Visceral fat area	195.3 [127.8, 266.5]	186.7 [119.9, 248.6]	0.26	188.1 [120.0, 260.0]
Subcutaneous fat area	553.8 [471.8, 621.0]	601.1 [431.6, 728.4]	0.23	593.2 [440.6, 703.2]
AST (U/L)	28.0 [19.0, 43.0]	23.0 [17.0, 36.5]	0.44	24.0 [17.5, 39.0]
ALT (U/L)	32.0 [12.0, 43.0]	32.0 [17.5, 44.0]	0.99	32.0 [16.8, 43.8]
Creatinine (mg/dL)	0.6 [0.5, 0.7]	0.7 [0.5, 0.8]	0.20	0.65 [0.53, 0.77]
eGFR (mL/min/1.73m^2^)	103.0 [90.5, 113.0]	92.0 [75.0, 107.0]	0.11	93.0 [80.3, 107.8]
HbA1c (%)	6.8 [6.2, 10.1]	6.7 [5.9, 9.5]	0.44	6.7 [6.0, 9.9]
Fasting blood glucose (mg/dL)	98.0 [96.0, 116.0]	105.0 [88.0, 127.0]	0.83	99.5 [88.0, 125.3]
Insulin (µIU/mL)	13.8 [8.6, 27.5]	13.9 [8.1, 18.5]	0.59	13.9 [8.5, 20.1 ]
CPR (ng/mL)	3.1 [2.3, 4.5]	3.1 [2.1, 3.8]	0.40	3.1 [2.1, 4.0]
HOMA-IR	5.7 [2.3, 6.7]	4.3 [2.1, 4.8]	0.56	4.7 [2.1, 5.0]
Natural log-transformed HS-CRP^a^	− 0.16 [− 0.62, − 0.01]	− 0.65 [− 0.95, − 0.25]	0.016	− 0.54 [− 0.87, − 0.14]
Diabetes^b^	12 (70.6%)	34 (72.3%)	1.00	46 (71.9%)
Dyslipidemia^b^	16 (94.1%)	30 (63.8%)	0.025	46 (71.9%)
Hyperuricemia^b^	15 (88.2%)	19 (40.4%)	< 0.001	34 (53.1%)
Hypertension^b^	14 (82.4%)	28 (59.6%)	0.26	44 (68.8%)

*IGF-1* Insulin like growth factor-1, *SDS* standard deviation scores, *GH* growth hormone, *BMI* body mass index, *AST* aspartate aminotransferase, *ALT* alanine aminotransferase, *eGFR* estimated glomerular filtration rate, *HbA1c* hemoglobin A1c, *CPR* connecting-peptide immunoreactivity, *HOMA*-IR homeostatic model assessment for insulin resistance, *HS-CRP* high-sensitivity C-reactive protein.

^a^Data was transformed as necessary to maintain assumptions of normality for the purposes of the analyses. ^b^Values are the number (%).

Otherwise, as shown in Table 1, the liver enzyme levels (aspartate aminotransferase (AST) and alanine aminotransferase (ALT) levels), parameters reflecting kidney function (including creatinine and eGFR) and glucose metabolism (including fasting plasma glucose, insulin levels, serum C-peptide immunoreactivity and the homeostatic model assessment for insulin resistance (HOMA-IR) did not differ between the two groups. Additionally, there were no differences in the parameters reflecting the hypothalamic–pituitary–adrenal (HPA), the hypothalamic–pituitary–thyroid (HPT), or the renin–angiotensin–aldosterone (RAA) axis (Supplementary Table 1), suggesting low IGF-1 levels to be minimally associated with dysregulation of other endocrinological axes. In addition, uric acids and lipid profiles, such as triglycerides, total cholesterol, high-density lipoprotein and low-density lipoprotein cholesterol levels, did not differ between the two groups (Supplementary Table 1). Similarly, atherosclerosis parameters, such as the ankle brachial index (ABI), cardio ankle vascular index (CAVI) and max intima-media thickness (IMT) were also similar in the two groups (Supplementary Table 1). On the other hand, interestingly, natural log-transformed high-sensitivity C-reactive protein (HS-CRP) was significantly elevated in the low IGF-1 group (− 0.16 [− 0.62, − 0.01] vs. − 0.65 [− 0.95, − 0.25] mg/dL, P = 0.016). Thus, low IGF-1 levels are linked to increased inflammation in obese subjects with BMI ≥ 35 kg/cm^2^.

We additionally analyzed the frequencies of commonly occurring obesity-related metabolic comorbidities (e.g. diabetes, dyslipidemia, hyperuricemia and hypertension) as shown in Table 1. We defined these metabolic comorbidities based on each of the relevant guidelines^21–24^. We also included patients who had a history and/or diagnosis of these metabolic disorders according to their medical records as having these comorbidities. The frequencies of dyslipidemia (16 (94.1%) vs. 30 (63.8%), *P* = 0.025) and hyperuricemia (15 (88.2%) vs. 19 (40.4%), *P* < 0.001) were significantly higher in the low than in the standard IGF-1 group. The frequency of hypertension tended to be high in the low IGF-1 group (14 (82.4%) vs. 28 (59.6%), *P* = 0.26), while that of diabetes was not increased (12 (70.6%) vs. 34 (72.3%), *P* = 1.00).

Collectively, these observations indicate that, despite their similar BMI, obese subjects with low IGF-1 levels are markedly more predisposed to have multiple metabolic abnormalities, such as increased adiposity, inflammation, dyslipidemia and hyperuricemia than subjects in the standard IGF-1 range.

### Increased adiposity, increased inflammation and high metabolic comorbidities were also confirmed in obese subjects who had low IGF-1 levels without diabetes

In addition to obesity, hyperglycemia and uncontrolled diabetes are applied as caveats when assessing GH-IGF-1 axis function in adults^1,8,25^. Since this study was conducted on inpatients managed at the Department of Diabetes and Metabolism in our hospital, the subjects included a high proportion (71.9%) of patients with diabetes. Therefore, we additionally conducted a similar analysis of the subjects without diabetes. We searched for any history of diabetes in clinical records and relied on the Japanese guideline^24^. Subjects already diagnosed as having diabetes on admission were also excluded.

**Figure 2:**
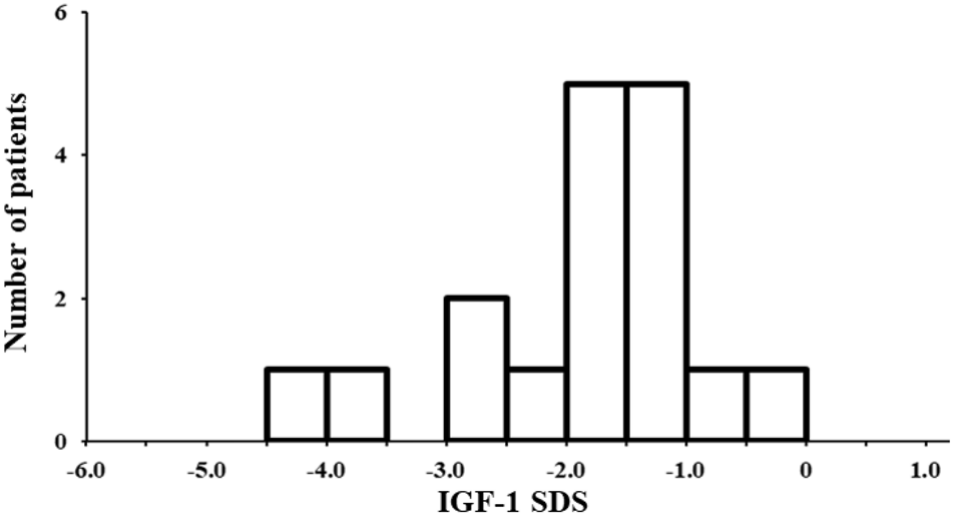
Histogram showing the number of the patients without diabetes per IGF-1 SDS category. *IGF-1* insulin like growth factor-1, *SDS* standard deviation score.

We found that the histogram reflecting the number of subjects without diabetes showed a similar shape with IGF-1 SDS values peaking around − 1.5. In addition, the percentage of low IGF-1 SDS subjects was 27.8% (Fig. 2), similar to that of the entire subjects (26.6%).

In the subjects without diabetes (Table 2), body fat percentage (53.5 [50.8, 53.5] vs. 45.1 [41.2, 48.0]%, *P* = 0.0083), blood glucose level (97.0 [97.0, 97.0] vs. 85.0 [82.0, 93.0] mg/dL, *P* = 0.038) and natural log-transformed HS-CRP (− 0.11 [− 0.17, − 0.01] vs. − 0.80 [− 1.02, − 0.30] mg/dL, *P* = 0.043) were significantly higher in the low IGF-1 than in the standard IGF-1 group. In addition, the frequency of dyslipidemia tended to be higher in the low IGF-1 group (5 (100.0%) vs. 7 (38.9%), *P* = 0.11). Notably, higher fasting glucose levels were identified as another significant parameter. In contrast, no significant differences were seen in the parameters reflecting the HPA, the HPT, or the RAA axis (Supplementary Table 2). These findings clearly suggest low IGF-1 levels to be associated with increased adiposity, inflammation and high comorbidities involving metabolic disorders in obese patients with BMI ≥ 35 kg/cm^2^, whether they have diabetes or not, independently from other endocrinological axes.

**Table 2:** Clinical characteristics of the obese subjects without diabetes according to the existence of low serumIGF-1 levels.

	Low IGF-1 group (n = 5)	Standard IGF-1 group (n = 13)	*P* value	All subjects (n = 18)
Sex (male/female)	1/4	7/6	0.31	8/10
Age (years)	30.0 [30.0, 37.0]	35.0 [28.0, 44.0]	0.62	34 [29.3, 39.0]
IGF-1 (ng/mL)	95.0 [95.0, 110.0]	128.0 [112.0, 146.0]	0.030	117.5 [97.8, 145.0]
IGF-1 SDS	− 2.8 [− 3.4, − 2.6]	− 1.4 [− 1.5, − 1.0]	0.0013	− 1.5 [− 2.1, − 1.2]
GH (ng/mL)	0.5 [0.4, 0.5]	0.4 [0.1, 1.2]	0.91	0.4 [0.3, 0.7]
Body weight on admission (kg)	105.7 [104.8, 110.6]	118.6 [110.0, 128.8]	0.35	111.2 [115.0, 128.0]
Body weight at discharge (kg)	100.6 [100.0, 108.6]	115.1 [99.8, 132.2]	0.59	106.3 [79.3, 129.5]
BMI (kg/m^2^)	42.1 [39.7, 46.3]	41.9 [38.7, 48.7]	0.66	42.0 [38.9, 48.3]
Systolic blood pressure (mmHg)	122.8 [107.0, 134.0]	134.0 [121.0、143.0]	0.26	130.9 [12.03, 142.8]
Diastolic blood pressure (mmHg)	76.0 [71.0, 84.0]	80.1 [77.0, 84.0]	0.55	78.9 [74.0, 84.0]
Body fat mass (kg)	57.6 [51.0, 59.0]	47.0 [43.3, 55.3]	0.19	51.0 [44.4, 57.6]
Body fat percentage (%)	53.5 [50.8, 53.5]	45.1 [41.2, 48.0]	0.0083	48.2 [44.2, 52.5]
Skeletal muscle mass index (kg/m^2^)	14.9 [12.8, 17.1]	12.1 [11.1, 13.3]	0.31	11.7 [10.8, 13.0]
AST (U/L)	13.0 [13.0, 19.0]	26.0 [16.0, 34.0]	0.13	21.0 [14.3, 33.8]
ALT (U/L)	11.0 [10.0, 12.0]	23.0 [18.0, 37.0]	0.068	21.0 [12.0, 36.8]
Creatinine (mg/dL)	0.6 [0.6, 0.7]	0.7 [0.7, 0.9]	0.14	0.7 [0.6, 0.8]
eGFR (mL/min/1.73m^2^)	93.0 [84.0, 103.0]	87.0 [77.0, 107.0]	0.43	89.0 [80.3, 106.0]
HbA1c (%)	6.1 [5.9, 6.2]	5.5 [5.5, 5.9]	0.18	5.9 [5.5, 6.1]
Fasting blood glucose (mg/dL)	97.0 [97.0, 97.0]	85.0 [82.0, 93.0]	0.038	90.0 [83.5, 97.0]
Insulin (µIU/mL)	17.0 [15.4, 28.7]	14.1 [9.8, 23.4]	0.52	16.0 [10.1, 23.9]
CPR (ng/mL)	3.4 [3.1, 4.5]	3.4 [2.8, 3.8]	0.52	3.4 [2.8, 3.8]
HOMA-IR	5.3 [3.7, 6.9]	3.7 [2.3, 4.9]	0.40	4.9 [2.1, 4.6]
Natural log-transformed HS-CRP^a^	− 0.11 [− 0.17, − 0.01]	− 0.80 [− 1.02, − 0.30]	0.043	− 0.58 [− 0.92, − 0.12]
Dyslipidemia^a^	5 (100.0)	7 (38.9)	0.11	12 (66.7)
Hyperuricemia^a^	3 (60.0)	6 (33.3)	1.00	9 (50.0)
Hypertension^a^	3 (60.0)	10 (55.6)	0.58	13 (72.2)

Because only one subject was conducted abdominal fat measurement in low IGF-1 group, we could not compare the data of visceral of subcutaneous fat area.

*IGF-1* insulin like growth factor-1, *SDS* standard deviation scores, *GH* growth hormone, *BMI* body mass index, *AST* aspartate aminotransferase, *ALT* alanine aminotransferase, *eGFR* estimated-glomerular filtration rate, *HbA1c* hemoglobin A1c, *CPR* connecting-peptide immunoreactivity, *HOMA*-IR homeostatic model assessment for insulin resistance, *HS*-*CRP* high-sensitivity C-reactive protein.

^a^Values are the number (%).

### The forward–backward stepwise variable selection procedure reveals adiposity to be a strong predictor of low IGF-1 levels

Next, using the entire subjects of this study, we aimed at identifying the factor most strongly associated with low IGF-1 levels among those showing statistically significant relevance (i.e. increased adiposity, dyslipidemia, hyperuricemia and increased inflammation). Four reliable variables associated with the low IGF-1 group (*P* < 0.05) (body fat percentage, dyslipidemia, hyperuricemia and natural log-transformed HS-CRP) were enrolled and we applied the forward–backward stepwise variable selection procedure. Body fat mass was excluded from this analysis because of strong correlation with body fat percentage. Based on the analysis, body fat percentage was solely selected (*P* = 0.0087), while other factors were eliminated (Table 3). When the analysis including HS-CRP instead of natural log-transformed HS-CRP was also performed, the same result showing that only body fat percentage was selected (*P* = 0.041) was obtained (Supplementary Table 3). Thus, increased adiposity is a more significant predictor of low IGF-1 levels than increased inflammation and multiple comorbidities. These findings suggest that low IGF-1 levels are primarily associated with increased adiposity, which may consequently exacerbate inflammation and induce multiple metabolic disorders.

**Table 3:** Results of the forward–backward stepwise selection procedure for the low IGF-1 group^a^.

Possible factors	Unadjusted odds ratio [95% CI]	Stepwise model	
		Wald-statistics	*P*-value
Body fat percentage (%)	1.34 [1.10–1.67]	6.89	0.0087
Natural log-transformed HS-CRP	1.69 [0.84–3.60]	–	–
Dyslipidemia (Ref; No)	9.07 [1.62–170.74]	–	–
Hyperuricemia (Ref; No)	11.05 [2.71–75.43]	–	–

*HS-CRP* high-sensitivity C-reactive protein.

^a^Forward-backward stepwise variable selection procedure was conducted with clinical parameters with P-value < 0.05 as the statistical significance entry criterion.

## Discussion

The first important finding in this study is that as high a proportion as 26.6% of patients with BMI ≥ 35 kg/cm^2^ had low IGF-1 levels (≤ − 2.0 SDS). This percentage is much higher than that in the general population (2.5%) based on its definition^11^. Many studies have demonstrated negative correlations between IGF-1 and BMI^6,13,14^, though conflicting results, i.e. normal or elevated IGF-1 levels in obese patients, were also reported^18^. This issue is further complicated by absolute IGF-1 values being affected by age and sex even in the healthy population^1^. Therefore, to reduce the confounding effects, IGF-1 SDS has recently been established for the evaluation of IGF-1 levels^10,11,25^. The SDS values readily allow us to compare IGF-1 levels between patients and the general population. Indeed, as noted in the guidelines and consensus statements^9,25,26^, the presence of dysfunction of the GH-IGF-1 axis is suspected when high (≥ + 2.0) or low (≤ − 2.0) IGF-1 SDS is detected in clinical practice. To our knowledge, this is the first reported study regarding the precise prevalence of low IGF-1 in obese subjects, to use IGF SDS (≤ − 2.0 SDS) as an evaluation tool.

We further clarified the clinical characteristics of obese subjects with low IGF-1 levels. By comparing the low and standard IGF-1 groups, obese subjects with low IGF-1 levels were revealed to be predisposed to increased adiposity, inflammation and metabolic disorders (dyslipidemia and hyperuricemia). In addition, in the subjects without diabetes, fasting hyperglycemia was found to be significantly linked to low IGF-1 levels, suggesting that low IGF-1 levels are associated with the pre-diabetic condition in obese population. Thus, multiple comorbidities involving metabolic disorders are important clinical characteristics of obese patients with low IGF-1 levels. To next identify the factor most strongly associated with low IGF-1 levels among increased adiposity, inflammation and high frequencies of dyslipidemia and hyperuricemia, we conducted stepwise variable selection. Employing this procedure, only increased adiposity is selected as a variable significantly associated with low IGF-1 levels. It is well known that obesity induces low-level chronic inflammation^27^, leading to multiple metabolic disorders related to obesity^28^. In the present study, notably, body fat mass and body fat percentage, but not BMI, were recognized as parameters significantly linked to low IGF-1 levels. Collectively, our observations suggest that the increased adiposity in obese patients may be a primary factor leading to high comorbidities involving multiple metabolic disorders.

What then is the mechanism underlying the strong association between low IGF-1 and increased adiposity? Several mechanisms acting at multiple steps, including decreased pituitary GH secretion due to decreased ghrelin secretion^16^ and suppressed IGF-1 production in the liver caused by obesity-related GH resistance^7,29^ may underlie the suppressed circulating IGF-1 levels associated with adiposity, although whether the alteration in hepatic IGF-BP production induced by hyperinsulinemia decreases or increases IGF-1 levels remains controversial^15,18^. On the other hand, while IGF-1 effects on adipocytes are still controversial, i.e. whether IGF-1 enhances lipolysis^30,31^ or not^32,33^, it is widely accepted that GH increases lipolysis through improved epinephrine-responsiveness in adipocytes^1,3,33,34^. Therefore, impairment of the GH-IGF-1 axis via decreased ghrelin might suppress lipolysis in obese subjects. These complex and bidirectional mechanisms may produce a vicious cycle, thereby forming a strong relationship between low IGF-1 levels and increased adiposity.

This study has several limitations. First, the statistical power is limited owing to the small sample size and the single-hospital setting. In particular, since we analyzed parameters in inpatients managed at the Department of Diabetes and Metabolism, the proportion of patients with diabetes was high. Notably, however, we obtained data indicating that the prevalence of those with low IGF-1 levels was similar (27.8%) in the subjects without diabetes, suggesting that the prevalence of those with low IGF-1 levels in the present study reflects obese subjects overall. Second, the study had a retrospective design. Prospective studies would provide more conclusive and long-term additional validation (e.g. more precise examination of time-courses of IGF-1 levels might herald future-increases in adiposity and the onsets of metabolic disorders). Especially, the future incidence rates of diabetes in the low IGF-1 group without diabetes may merit investigation. Third, we did not measure serum IGF-1 binding protein (IGF-1BP). The biological activity of IGF-1 is generally known to be regulated through six types of IGF-1 BPs. IGF-1 has higher affinity for IGF-1 BP than the IGF-1 receptor and its binding to IGF-1 BP produces inactive forms, while free IGF-1 is known to be bioactive IGF-1^3,8,35–37^. In clinical practice, however, IGF-1 measurement shows total IGF-1 including free and inactivated IGF-1^38^ including the assays in this study (BML Corporation, Tokyo, Japan). Therefore, we analyzed correlations of serum IGF-1 levels, rather than its activity, with several metabolic parameters. Fourth, we did not perform GH-stimulation tests to evaluate the pituitary capacity of GH secretion. Therefore, whether or not the low IGF-1 levels observed in obesity subjects are attributable to decreased GH secretion in the pituitary remains to be elucidated. Finally, liver functions may have exerted influences on our observations. Although we excluded patients with hepatic cirrhosis from this study, liver-produced IGF-1 can be affected by the conditions of nonalcoholic fatty liver disease^39,40^. However, since the liver enzyme (AST and ALT) levels were similar in the standard and low IGF-1 groups in this study, we suspected that such influences would have been minimal.

In conclusion, we revealed that as much as 26.6% of patients with BMI ≥ 35 kg/m^2^ had low IGF-1 levels, making this the first report to demonstrate the prevalence of low IGF-1 SDS levels in obese patients. We also revealed clinical characteristics of obese patients with low IGF-1 levels, i.e. increased adiposity and inflammation and metabolic comorbidities, such as dyslipidemia and hyperuricemia. Additionally, low IGF-1 levels were associated with high fasting glucose levels in subjects without diabetes, suggesting that IGF-1 levels are a potential marker of pre-diabetes in obese patients. We should be cautious in interpreting and managing IGF-1 levels, as a parameter for evaluating the risks of metabolic disorders, in obese subjects.

## Methods

### Patient population and data collection

We retrospectively searched the clinical records of Japanese patients 20 to 77 years of age with obesity (BMI ≥ 35 kg/m^2^) hospitalized to receive medical treatment at our department in Tohoku University Hospital between April 2013 and October 2019, since BMI ≥ 35 kg/m^2^ is generally accepted as being a risk factor for adverse health outcomes in the Japanese population^19,20^. The patients had been admitted for diet and exercise therapy with the aim of achieving weight loss. No patients had been clinically managed with very low-calorie diets or ketogenic diets prior to admission. There were 166 subjects who met the above screening criteria. Thereafter, subjects meeting the following criteria were excluded from this study (n = 42) due to the possibility of having an additional influence on IGF-1 levels; (1) past history of malignancy (n = 19), (2) thyroid dysfunction (n = 12), (3) liver cirrhosis (n = 3), (4) pre-diagnosed congenital or secondary GH deficiency (GHD), such as Prader-Willi syndrome and pituitary diseases (n = 6) and (5) out-of-age-range (age < 18 years) for the IGF-1 SDS conversion^11^ (n = 2). In addition, subjects in whom serum IGF-1 levels had not been measured while in resting positions (n = 60) were also excluded. Finally, we analyzed the subjects with severe obesity who had not met these exclusion criteria as the research target group (n = 64). The retrospective and single-centered study protocol was approved by the Research Ethics Committee of Tohoku University (no. 2019-1-976), which waived the requirement for written informed consent due to the use of anonymized data obtained as part of regular medical examinations. We also followed the practice of the Declaration of Helsinki and relevant guidelines, regulations and policies as required by the journal.

### Definitions of hypertension, hyperuricemia, dyslipidemia and diabetes

According to the Japanese guideline for hypertension^21^, individuals with at least one of the following findings are diagnosed as having hypertension: (1) office blood pressure levels of 140/90 mmHg or more and/or (2) out-of-office blood pressure levels of 135/85 mmHg or more. We also regarded subjects as having hypertension if they were taking antihypertensive medications during hospitalization. Hyperuricemia is defined as serum uric acid levels of more than 7.0 mg/dL according to the Japanese guideline^22^. We also considered subjects who had already been taking medication for hyperuricemia during hospitalization to meet this definition. Dyslipidemia is defined according to the Japan Atherosclerosis Society (JAS) guidelines^23^ as having at least one of the following findings; (1) elevated low-density lipoprotein (LDL) cholesterol ≥ 140 mg/dl, (2) high-density lipoprotein (HDL) cholesterol < 40 mg/dL, (3) triglycerides ≥ 150 mg/dL and/or (4) non-HDL cholesterol ≥ 170 mg/dL in fasting states. We directly measured LDL cholesterol in our hospital laboratory. The Friedewald formula was not used. We also considered individuals who were already taking medications for dyslipidemia as having this condition. Diabetes was diagnosed based on the Japanese guideline^24^, as follows. First, subjects were judged as exhibiting the diabetic type if they met one of the following criteria: (1) fasting plasma glucose level of ≥ 126 mg/dL (≥ 7.0 mmol/L), (2) 2-h value of ≥ 200 mg/dL (≥ 11.1 mmol/L) on 75 g oral glucose tolerance test (OGTT), (3) casual plasma glucose level of ≥ 200 mg/dL (≥ 11.1 mmol/L), or (4) hemoglobin A1c (HbA1c) based on the national glycohemoglobin standardization program ≥ 6.5%. Otherwise, if the subject met any one or more of the three criteria for plasma glucose values (1, 2 and 3) and HbA1c (4) using the same blood sample, diabetes was diagnosed based on the initial examination alone. Any patient who did not meet these criteria, was re-examined on another day. If his/her diabetic type was reconfirmed, the patient was diagnosed as having diabetes mellitus. However, a diagnosis could not be made based solely on the re-examination of HbA1c. We also included patients already taking anti-diabetic medications among those with this diagnosis.

We additionally defined patients as having these diseases if they had already been diagnosed at other hospitals, or based on their clinical records prior to the current admission, because laboratory data obtained at one point in time might be affected by medications or dietary treatment. Patients whose diagnosis was confirmed during hospitalization in this study were also included in the groups with these diseases.

### Biochemical analysis and IGF-1 SD score determination

Blood samples were obtained in the resting position in the morning (routine blood specimens were ordered to be drawn at 6:00 am) for a fasted blood sample on the second day of admission. Serum creatinine was assessed using standard enzymatic methods. The estimated glomerular filtration rate (eGFR) was calculated using the formula recommended by the Japanese Society of Nephrology, derived from the Modification of Diet in Renal Disease (MDRD) study group^41^. Hemoglobin A1c was assayed using high-performance liquid chromatography and was expressed as National Glycohemoglobin Standardization Program units. Serum insulin and C-peptide were measured using an electro-chemiluminescent immunoassay (ECLIA). HS-CRP was assessed using a latex-enhanced turbidimetric immunoassay. Hormonal levels were measured using the following commercially available products: IGF-1: immunoradiometric assay (IRMA) until March 2019 and ECLIA after April 2019 (BML Corporation, Tokyo, Japan), GH; chemiluminescent immunoassay (CLIA) until March 2017 and ECLIA after April 2017 (BML Corporation, Tokyo, Japan). All other assays are described in each of the relevant legends. Insulin resistance was calculated using the HOMA-IR model [HOMA-IR = fasting insulin (µIU/mL)*fasting glucose (mmol/L)/22.5]^42^. IGF-1 SDS, adjusted by age and sex, was calculated as previously reported^11^ and the ECLIA values were converted to the values of IRMA provided by the BML Corporation. GH values calculated employing ECLIA were similarly converted to the CLIA values. For this analysis, we defined the “low IGF-1 group” as the patients with IGF-1 levels of − 2.0 SDS or less and “standard IGF-1 group” as more than − 2.0 SDS. There were no patients whose IGF-1 SDS levels exceeded + 2.0 SDS.

### Assessment of body composition

Body fat and skeletal muscle masses were measured employing bioelectrical impedance analysis using InBody 770 (InBody Japan Co., Ltd., Tokyo, Japan). Abdominal subcutaneous and total fat areas were measured with CT scans at the fourth lumbar vertebral level applying the SOMATOM Definition (Siemens AG., Munich, Germany). The intra-abdominal cavity area was outlined on CT. The visceral fat area was measured at an attenuation range of − 200 and − 50 Hounsfield units^43^.

### Cardio-ankle vascular index and ankle-brachial index measurements

ABI and CAVI were measured using VaSera VS-1500 or 3000 (Fukuda Denshi, Tokyo, Japan). In brief, patients rested for 5 min, and their blood pressures were then measured at both upper arms and ankles using an oscillometric method to obtain the ABI and CAVI. The averages of the right and left ABI and CAVI values were used for the analyses.

### Carotid ultrasonographic measurements

Well-trained ultra-sonographers examined the carotid arteries using Aplio i900 (Canon medical, Tokyo, Japan), Aplio 500 (Toshiba Corporation, Tokyo, Japan) and ProSound F75 (Hitachi-Aloka, Tokyo, Japan) devices. The maximum intima-media thickness (max-IMT) was measured in the left and right common carotid arteries as described previously^44^. The averages of the right and left max-IMT values were used for the analyses.

### Statistical analysis

Continuous variables are shown as medians (interquartile ranges) given their non-standard distributions. Other data are presented as a number (%) unless otherwise indicated. The differences in clinical parameters between the low IGF-1 and standard IGF-1 groups were analyzed using the Mann–Whitney *U* test for continuous variables or the Fisher exact test for categorical variables. The forward–backward stepwise variable selection procedure was performed with a P-value < 0.05 as the statistical significance entry level for clarifying explanatory clinical parameters. Estimates of odds ratios [95% confidence intervals (CIs)] are presented for interquartile range (IQR) increases in these parameters. The analyses were performed using JMP software version 12.0.1 (SAS Institute Inc., Cary, NC, USA). *P* < 0.05 was considered to indicate a statistically significant difference.

## Supplementary Information

Supplementary Tables.

## Data Availability

The datasets used and/or analyzed during the current study are available from the corresponding author on reasonable request.
