# Supplementary material for: Insulin-like growth factor-1 levels are associated with high comorbidity of metabolic disorders in obese subjects; a Japanese single-center, retrospective-study
Source: Sci Rep. 2022 Nov 22;12:20130. doi: 10.1038/s41598-022-23521-1 (PMC9684525; doi:10.1038/s41598-022-23521-1)
Supplement: Supplementary file 1 — Supplementary Tables. [file 41598_2022_23521_MOESM1_ESM.pdf]

Insulin-Like Growth Factor-1 Levels Are Associated with High Comorbidity of Metabolic Disorders in Obese Subjects; a Japanese Single-Center, Retrospective-Study

Haremaru Kubo[1], Shojiro Sawada[1,2], Michihiro Satoh[3], Yoichiro Asai[1], Shinjiro Kodama[1], Toshihiro Sato[1], Seitaro Tomiyama[1], Junro Seike[1], Kei Takahashi[1], Keizo Kaneko[1], Junta Imai[1], and Hideki Katagiri[1]

[1]Department of Diabetes and Metabolism, Tohoku University Hospital, Sendai, Japan

[2]Division of Metabolism and Diabetes, Faculty of Medicine, Tohoku Medical and Pharmaceutical University, Sendai, Japan

[3]Division of Public Health, Hygiene and Epidemiology, Faculty of Medicine, Tohoku Medical and Pharmaceutical University, Sendai, Japan

Supplementary digital content files: 3 tables

Correspondence:

Hideki Katagiri, MD, PhD.

Department of Metabolism and Diabetes, Tohoku University Graduate School of Medicine, Sendai, Miyagi, Japan.

Tel: +81-22-717-7611

Fax: +81-22-717-7179

e-mail: katagiri@med.tohoku.ac.jp

	low IGF-1 group (n=17)	standard IGF-1 group (n=47)	*P* value	all group (n=64)
TSH (mIU/mL)[a]	1.80 [1.16, 3.34]	1.92 [1.20, 2.71]	0.70	1.86 [1.18, 2.71]
FT4 (ng/mL)[a]	1.14 [1.08, 1.19]	1.22 [1.12, 1.39]	0.16	1.17 [1.06, 1.33]
FT3 (pg/mL)[a]	2.71 [2.70, 3.01]	2.90 [2.72, 3.26]	0.30	2.90 [2.71, 3.20]
PRA (ng/mL/hr)[b]	2.1 [1.3, 4.1]	1.8 [1.0, 3.5]	0.37	1.9 [1.1, 3.8]
PAC (ng/mL)[b]	9.3 [7.0, 10.8]	8.7 [7.4, 11.0]	1.00	8.7 [7.3, 11.0]
ACTH (pg/mL)[b]	31.9 [15.6, 41.7]	37.5 [16.3, 54.2]	0.32	36.2 [16.3, 52.0]
Cortisol (μg/dL)[b]	8.2 [2.7, 14.2]	12.5 [8.7, 16.0]	0.10	11.9 [6.3, 15.9]
Triglyceride (mg/dL)[c]	121.0 [103.0, 187.0]	119.0 [91.0, 173.0]	0.58	120.0 [91.8, 176.5]
Total choresterol (mg/dL)[c]	183.0 [156.0, 191.0]	176.0 [158.0, 206.5]	0.90	179.5 [156.8, 206.0]
HDL choresterol (mg/dL)[c]	44.0 [36.0, 48.0]	39.0 [36.0, 45.0]	0.20	43.0 [37.0, 48.0]
LDL choresterol (mg/dL)[c]	113.0 [99.0, 133.0]	112.0 [96.0, 134.0]	0.70	112.5 [96.5, 134.0]
Uric Acid (mg/dL)[c]	5.8 [5.0, 7.0]	6.5 [5.8, 7.6]	0.09	6.3 [5.6, 7.2]
Systolic blood pressure (mmHg)	136.5 [125.5, 146.5]	133.6 [122.0, 146.0]	0.60	135.7 [124.5, 146.0]
Diastolic blood pressure (mmHg)	83.2 [74.0、97.0]	84.2 [77.0, 90.0]	0.80	83.9 [77.0, 91.5]
max-IMT (mm)	0.7 [0.6, 0.9]	0.7 [0.6, 1.0]	0.69	0.7 [0.6, 0.9]
ABI	1.04 [0.98, 1.08]	1.02 [0.94, 1.10]	0.92	1.02 [0.96, 1.10]
CAVI	5.8 [5.4, 6.2]	6.1 [5.2, 7.3]	0.29	6.0 [5.2, 7.1]

Supplementary Table 1. Clinical characteristics of other endocrinological and metabolic data in obese patients in low IGF-1 and standard IGF-1 groups.

[a]Thyroid function tests including thyroid stimulating hormone (TSH), free T4 (FT4; thyroxine) and free T3 (FT3; tri-iodothyronine) were measured by ECLIA.

[b]Other hormonal evaluations were measured using followings commercially: Plasma renin activity (PRA): enzyme immunoassay (EIA) (LSI Medience Corporation, Tokyo, Japan), Plasma aldosterone concentration (PAC): radioimmunoassay (RIA) (LSI Medicine Corporation, Tokyo, Japan), ACTH: ECLIA (LSI Medicine Corporation, Tokyo, Japan), Cortisol: chemiluminescence immunoassay (Siemens Healthcare Diagnostic, Tokyo, Japan).

[c]Serum triglyceride, total cholesterol, high-density lipoprotein (HDL) cholesterol, low-density lipoprotein (LDL) cholesterol and serum uric acid levels were assessed using standard enzymatic methods.

Abbreviations: TSH, thyroid stimulating hormone; FT4, free T4 (T4; thyroxine); FT3, free T3 (T3, tri-iodothyronine); PRA, plasma renin activity; PAC, plasma aldosterone concentration; HDL, high density lipoprotein; LDL, low density lipoprotein; IMT, intima media thickness; ABI, ankle brachial pressure index; CAVI, cardio-ankle vascular index

	low IGF-1 group (n=5)	standard IGF-1 group (n=13)	*P* value	all group (n=18)
TSH (mIU/mL)[a]	1.37 [0.75, 2.83]	2.29 [1.31, 4.69]	0.37	2.20 [1.18, 4.69]
FT4 (ng/mL)[a]	1.16 [1.06, 1.32]	1.39 [1.26, 1.48]	0.24	1.35 [1.18, 1.48]
FT3 (pg/mL)[a]	2.91 [2.64, 2.93]	3.41 [2.91. 3.49]	0.21	2.97 [2.90, 3.42]
PRA (ng/mL/hr)[b]	3.0 [2.1, 3.8]	1.2 [0.8, 1.5]	0.16	1.2 [1.0, 2.1]
PAC (ng/mL)[b]	10.8 [9.5, 60.4]	8.7 [7.8, 11.0]	0.40	8.7 [81, 11.0]
ACTH (pg/mL)[b]	17.7 [11.1, 34.7]	39.8 [9.2, 57.1]	0.47	38.2 [8.5, 53.3]
Cortisol (μg/dL)[b]	3.6 [1.4, 8.2]	12.5 [3.8, 15.6]	0.27	11.5 [2.4, 15.5]
Triglyceride (mg/dL)[c]	118.0 [103.0, 121.0]	87.0 [74.0, 122.0]	0.30	95.5 [77.5, 121.8]
Total choresterol (mg/dL)[c]	180.0 [169.0, 183.0]	172.0 [151.0, 184.0]	0.73	173.5 [153.3, 183.8]
HDL choresterol (mg/dL)[c]	38.0 [38.0, 41.0]	45.0 [42.0, 47.0]	0.35	40.0 [35.3, 45.8]
LDL choresterol (mg/dL)[c]	113.0 [99.0, 125.0]	110.0 [100.0, 129.0]	0.96	111.0 [99.3, 128.0]
Uric Acid (mg/dL)[c]	6.4 [5.8, 7.7]	6.7 [5.9, 7.9]	0.66	6.7 [5.8, 7.9]
Systolic blood pressure (mmHg)	122.8 [107.0, 134.0]	134.0 [121.0、143.0]	0.26	130.9 [12.03, 142.8]
Diastolic blood pressure (mmHg)	76.0 [71.0, 84.0]	80.1 [77.0, 84.0]	0.55	78.9 [74.0, 84.0]
max-IMT (mm)	0.7 [0.6, 0.7]	0.7 [0.5, 0.7]	0.82	0.7 [0.5, 0.7]
ABI	0.97 [0.92, 0.98]	0.98 [0.93, 1.10]	0.36	0.98 [0.93, 1.02]
CAVI	6.0 [5.6, 6.3]	5.0 [4.7, 5.3]	0.17	5.2 [4.8, 6.0]

Supplementary Table 2. Clinical characteristics of other endocrinological and metabolic data in obese patients without diabetes in low IGF-1 and standard IGF-1 groups.

[a]Thyroid function tests including thyroid stimulating hormone (TSH), free T4 (FT4; thyroxine) and free T3 (FT3; tri-iodothyronine) were measured by ECLIA.

[b]Other hormonal evaluations were measured using followings commercially: Plasma renin activity (PRA): enzyme immunoassay (EIA) (LSI Medience Corporation, Tokyo, Japan), Plasma aldosterone concentration (PAC): radioimmunoassay (RIA) (LSI Medicine Corporation, Tokyo, Japan), ACTH: ECLIA (LSI Medicine Corporation, Tokyo, Japan), Cortisol: chemiluminescence immunoassay (Siemens Healthcare Diagnostic, Tokyo, Japan).

[c]Serum triglyceride, total cholesterol, high-density lipoprotein (HDL) cholesterol, low-density lipoprotein (LDL) cholesterol and serum uric acid levels were assessed using standard enzymatic methods.

Abbreviations: TSH, thyroid stimulating hormone; FT4, free T4 (T4; thyroxine); FT3, free T3 (T3, tri-iodothyronine); PRA, plasma renin activity; PAC, plasma aldosterone concentration; HDL, high density lipoprotein; LDL, low density lipoprotein; IMT, intima media thickness; ABI, ankle brachial pressure index; CAVI, cardio-ankle vascular index

Possible Factors	Unadjusted Odds Ratio [95%CI]	Stepwise Model	
		Wald-statistics	*P*-value
Body fat percentage (%)	1.34 [1.10-1.67]	4.16	0.041
HS-CRP	0.11 [0.0047-2.05]	-	-
Dyslipidemia (Ref; No)	9.07 [1.62-170.74]	-	-
Hyperuricemia (Ref; No)	11.05 [2.71-75.43]	-	-

Supplementary Table 3. Results of stepwise selection procedure for low IGF-1 group using HS-CRP [a].

[a]Forward-backward stepwise variable selection procedure to clarify clinical parameters with P-value <0.05 as statistical significance entry criteria.

Abbreviation: HS-CRP, high-sensitivity C-reactive protein.
